# The calorically restricted ketogenic diet, an effective alternative therapy for malignant brain cancer

**DOI:** 10.1186/1743-7075-4-5

**Published:** 2007-02-21

**Authors:** Weihua Zhou, Purna Mukherjee, Michael A Kiebish, William T Markis, John G Mantis, Thomas N Seyfried

**Affiliations:** 1 Department of Biology, Boston College, Chestnut Hill, USA

## Abstract

**Background:**

Malignant brain cancer persists as a major disease of morbidity and mortality in adults and is the second leading cause of cancer death in children. Many current therapies for malignant brain tumors fail to provide long-term management because they ineffectively target tumor cells while negatively impacting the health and vitality of normal brain cells. In contrast to brain tumor cells, which lack metabolic flexibility and are largely dependent on glucose for growth and survival, normal brain cells can metabolize both glucose and ketone bodies for energy. This study evaluated the efficacy of KetoCal^®^, a new nutritionally balanced high fat/low carbohydrate ketogenic diet for children with epilepsy, on the growth and vascularity of a malignant mouse astrocytoma (CT-2A) and a human malignant glioma (U87-MG).

**Methods:**

Adult mice were implanted orthotopically with the malignant brain tumors and KetoCal^® ^was administered to the mice in either unrestricted amounts or in restricted amounts to reduce total caloric intake according to the manufacturers recommendation for children with refractory epilepsy. The effects KetoCal^® ^on tumor growth, vascularity, and mouse survival were compared with that of an unrestricted high carbohydrate standard diet.

**Results:**

KetoCal^® ^administered in restricted amounts significantly decreased the intracerebral growth of the CT-2A and U87-MG tumors by about 65% and 35%, respectively, and significantly enhanced health and survival relative to that of the control groups receiving the standard low fat/high carbohydrate diet. The restricted KetoCal^® ^diet reduced plasma glucose levels while elevating plasma ketone body (β-hydroxybutyrate) levels. Tumor microvessel density was less in the calorically restricted KetoCal^® ^groups than in the calorically unrestricted control groups. Moreover, gene expression for the mitochondrial enzymes, β-hydroxybutyrate dehydrogenase and succinyl-CoA: 3-ketoacid CoA transferase, was lower in the tumors than in the contralateral normal brain suggesting that these brain tumors have reduced ability to metabolize ketone bodies for energy.

**Conclusion:**

The results indicate that KetoCal^® ^has anti-tumor and anti-angiogenic effects in experimental mouse and human brain tumors when administered in restricted amounts. The therapeutic effect of KetoCal^® ^for brain cancer management was due largely to the reduction of total caloric content, which reduces circulating glucose required for rapid tumor growth. A dependency on glucose for energy together with defects in ketone body metabolism largely account for why the brain tumors grow minimally on either a ketogenic-restricted diet or on a standard-restricted diet. Genes for ketone body metabolism should be useful for screening brain tumors that could be targeted with calorically restricted high fat/low carbohydrate ketogenic diets. This preclinical study indicates that restricted KetoCal^® ^is a safe and effective diet therapy and should be considered as an alternative therapeutic option for malignant brain cancer.

## Background

Malignant brain cancer persists as a major disease of morbidity and mortality in adults and is the second leading cause of cancer death in children [1-4]. Many current therapies for malignant brain tumors are ineffective in providing long-term management because they focus on the defects of the tumor cells at the expense of the health and vitality of normal brain cells [5-7]. We previously showed that caloric restriction (CR) is anti-angiogenic, anti-inflammatory, and pro-apoptotic in the experimental mouse (CT-2A astrocytoma) and the human (U87-MG malignant glioma) brain tumors [8-11]. CR targets tumor cells by reducing circulating glucose levels and glycolysis, which tumor cells need for survival, and by elevating ketone bodies, which provide normal brain cells with an alternative fuel to glucose [5,9,11]. We previously used linear regression analysis to show that blood glucose levels could predict CT-2A growth as well as insulin-like growth factor 1 (IGF-1) levels, which influences tumor angiogenesis [8,9]. In contrast to glucose, ketone bodies (β-hydroxybutyrate and acetoacetate) bypass glycolysis and directly enter the mitochondria for oxidation [12,13]. By bypassing glycolysis, ketone bodies are also effective for treatment of inherited defects in glucose transporters and pyruvate dehydrogenase, which connects glycolysis with respiration [14-16]. Ketone bodies are more energetically efficient than either pyruvate or fatty acids because they have a greater hydrogen/carbon ratio (more reduced) than pyruvate and, unlike fatty acids, do not uncouple mitochondria [5,17]. The transition from glucose to ketone bodies for brain energy metabolism is best under the natural conditions of CR [5,8,18].

The metabolism of β-hydroxybutyrate (β-OHB), the major circulating ketone body, for energy depends on the expression of two key mitochondrial enzymes: β-hydroxybutyrate dehydrogenase (β-OHBDH), and succinyl-CoA: 3-ketoacid CoA transferase (SCOT) [13,19-21]. These enzymes become critical when neurons and glia transition to ketone bodies in order to maintain energy balance under conditions of reduced glucose availability. In addition to serving as a more efficient metabolic fuel than glucose, ketone bodies also possess anti-inflammatory potential through reduction of reactive oxygen species and increase of glutathione peroxidase activity [5,17,22]. Brain tumors, like most malignant tumors, are largely dependent on glucose and glycolysis for their growth and survival due to abnormalities in the number and function of their mitochondria [5,8,23-27]. The transition from glucose to ketone bodies as the primary energy source of the brain under calorically restricted conditions exploits the metabolic deficiencies of brain tumor cells while enhancing the health and vitality of normal neurons and glia according to principles of evolutionary biology and metabolic control theory [5,28].

Nebeling and co-workers previously found that a high fat/low carbohydrate ketogenic diet (KD), consisting of medium chain triglycerides, provided long-term management of pediatric astrocytoma while enhancing the nutritional status of the patients [29]. The findings in human pediatric astrocytoma were confirmed in the experimental mouse CT-2A astrocytoma using a lard-based rodent ketogenic diet [8,9]. CR and some KDs, however, are not standardized diets and may be difficult to implement in the clinic due to issues of compliance. For example, CR in mice mimics therapeutic fasting in humans, involving water-only dieting, whereas medium chain triglyceride or lard-based ketogenic diets can cause gastrointestinal and kidney problems in both children and adults [30-33]. Our goal was to develop a more effective alternative diet therapy for brain cancer that could extend survival without compromising the health and vitality of normal cells.

In this study we evaluated the therapeutic efficacy of KetoCal^®^, a new nutritionally balanced soybean oil ketogenic diet that was formulated specifically for managing refractory epilepsy in children [34]. No prior studies have evaluated the therapeutic efficacy of KetoCal^® ^for brain cancer management. Here we show that KetoCal^®^, given in calorically restricted amounts significantly reduced circulating plasma glucose levels while significantly elevating ketone body levels in mice bearing orthotopic CT-2A and U87-MG brain tumors. Moreover, the restricted KetoCal^® ^diet reduced brain tumor growth and microvessel density, while extending mouse survival. Gene expression for β-OHBDH and SCOT was lower in the tumors than in contralateral normal brain suggesting that the brain tumors have reduced ability to metabolize ketone bodies for energy. This preclinical study indicates that KetoCal^® ^is a safe and effective diet therapy for malignant brain cancer and can be considered as an alternative or adjuvant therapeutic option. A preliminary report on this work has appeared [35].

## Methods

### Mice

Mice of the C57BL/6J (B6) strain and the BALBc/J-severe combined immunodeficiency (SCID) strain were obtained from the Jackson Laboratory, Bar Harbor, ME. The mice were propagated in the animal care facility of the Department of Biology, Boston College, using animal husbandry conditions described previously [36]. Male mice (10–12 weeks of age) were used for the studies and were provided with food under either restricted or unrestricted conditions (as below). Water was provided *ad libitum *to all mice. The SCID mice were maintained in laminar flow hoods in a pathogen free environment. All animal experiments were carried out with ethical committee approval in accordance with the National Institutes of Health Guide for the Care and Use of Laboratory Animals and were approved by the Institutional Animal Care Committee.

### Brain tumor models

The syngeneic mouse brain tumor CT-2A, was originally produced by implantation of a chemical carcinogen, 20-methylcholanthrene, into the cerebral cortex of B6 mice and was characterized as an anaplastic astrocytoma [37,38]. The morphological, biochemical, and growth characteristics of the CT-2A mouse brain tumor were previously described [37,39-42]. The U87-MG (U87) tumor was originally derived from a human malignant glioma cell line and was grown as a xenograft in the SCID mice [43,44].

### Intracerebral and subcutaneous tumor implantation

The mouse CT-2A and human U87 tumors were implanted into the cerebral cortex of the B6 or SCID mice, respectively, using a trocar as we previously described [41]. Briefly, mice were anesthetized with 2,2,2-tribromoethanol (Sigma Aldrich, St. Louis, MO) given intra-peritoneally and their heads were shaved and swabbed with 70% ethyl alcohol under sterile conditions. Small tumor pieces (about 1 mm^3^) from donor mice were implanted into the right cerebral hemisphere of anesthetized recipient mice as we recently described [41]. Initiation of tumors from intact tumor pieces is preferable to initiation from cultured cells, since the tumor tissue contains an already established microenvironment that facilitates rapid tumor growth [41]. All of the mice recovered from the surgical procedure and were returned to their cages when they appeared fully active.

For the survival studies, CT-2A or U87 flank-grown tumors were removed from B6 or SCID mice, respectively, and were rinsed and diced in cold phosphate-buffered saline (PBS) at pH 7.4. Mice were anesthetized with isoflurane (Halocarbon, NJ) and 0.1 ml of diced tumor tissue, suspended in 0.2 ml PBS, was implanted subcutaneously (s.c.) in the right flank by injection using a 1 cc tuberculin syringe and 18-gauge needle. The flanks of recipient mice were shaved in order to facilitate tumor detection and growth assessment. The inoculated mice were monitored daily for nodule formation.

### Diets and feeding

The mice were group housed prior to the initiation of the experiment and were then individually housed in plastic shoebox cages one day before tumor implantation. All mice received PROLAB chow (Agway Inc., NY) prior to the experiment. This is a standard high carbohydrate mouse chow diet (SD) and contains a balance of mouse nutritional ingredients. According to the manufacturers specification, this diet delivers 4.4 Kcal/g gross energy, where fat, carbohydrate, protein, and fiber comprised 55 g, 520 g, 225 g, and 45 g/Kg of the diet, respectively. The KetoCal^® ^ketogenic diet was obtained as a gift from Nutricia North America (Rockville, MD, formally SHS International, Inc.). KetoCal^® ^is a nutritionally complete ketogenic formula and, according to the manufacturers specification, delivers 7.2 Kcal/g gross energy where fat, carbohydrate, protein, and fiber comprised 720 g, 30 g, 150 g, and 0 g/Kg of the diet, respectively. There are also minor differences between the two diets for the content (g/kg of diet) of amino acids, vitamins, minerals and trace elements. The diet has a ketogenic ratio (fats: proteins + carbohydrates) of 4:1 and the fat was derived from soybean-oil. The KetoCal^® ^diet was fed to the mice in paste form (water: KetoCal^®^; 1:2) within the cage using procedures as we previously described [18]. A comparison of the nutritional composition of the SD and the KetoCal^® ^diet is presented in Table 1.

**Table 1:** Composition (%) of the Standard Diet and the KetoCal^® ^Ketogenic diet^a^

**Components**	**Standard Diet (SD)**	**KetoCal^®^Diet (KC)**
Carbohydrate	65	3.3
Fat	6.9	80
Protein	28.1	16.7
Energy (Kcal/gr)	4.4	7.2

^a ^Values obtained from normalizing energy content (see Methods)

**Figure 1:**
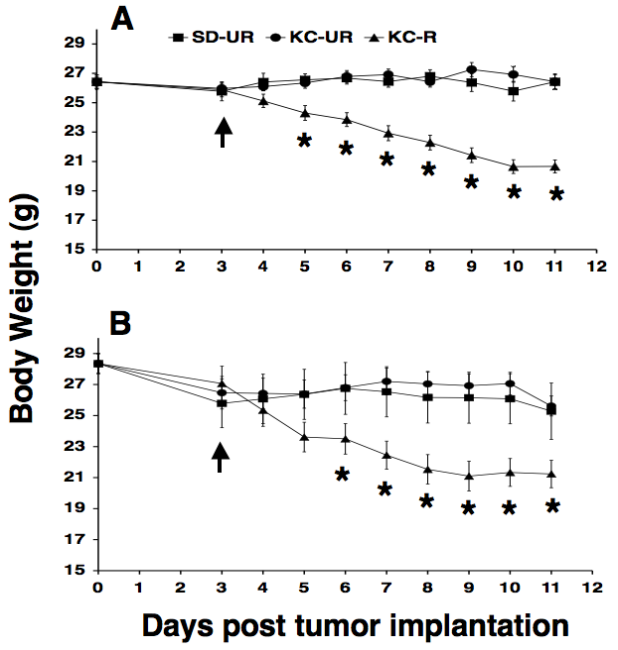
Influence of diet on body weight in B6 mice bearing the mouse CT-2A astrocytoma (A), and in SCID mice bearing the human U87 glioma (B). Tumors were implanted orthotopically at day 0, and diets were initiated at day 3 (arrow). The SD-UR, KC-UR, and KC-R represent the standard high carbohydrate diet fed unrestricted, the KetoCal^® ^diet fed unrestricted, and the KetoCal^® ^diet fed in restricted amounts, respectively. Values are expressed as the mean ± SEM (n = 12–14 mice per group), and asterisks indicate that the body weights of the KC-R mice (▴) were significantly lower than those of the unrestricted control groups at P < 0.01.

The animal room was maintained at 22 ± 1°C, and cotton nesting pads were provided for additional warmth. All intracerebral (i.c.) tumor-bearing B6 and SCID mice were maintained on the SD for three days following tumor implantation (day 0, Figure 1) and were then randomly assigned (arrow, Figure 1) to one of three diet groups that received either: 1) the standard diet fed *ad libitum*, or unrestricted (SD-UR), 2) the KetoCal^® ^diet fed *ad libitum*, or unrestricted (KC-UR), 3) the KC diet restricted to reduce body weight by approximately 20% of the original body weight at day 3 (KC-R). The dietary treatments were continued for 8 days for tumor weight analysis. For the survival analysis, the dietary treatment was initiated 7 days after subcutaneous implantation and continued until the tumors reached 2.5 cm^3^.

### Tumor growth and survival analysis

Intracerebral tumor growth was analyzed directly by measuring total tumor wet weight. Tumors were dissected from normal appearing brain tissue and were weighed. Survival was determined as the time for subcutaneous tumors to reach the size of 2.5 cm^3 ^after tumor inoculation as previously described [45]. Calipers were used to measure tumor volume as previously described [46]. Tumor nodules appeared approximately 7 days following inoculation.

### Measurement of plasma glucose and β-hydroxybutyrate (β-OHB)

Blood was collected from mice on the last day of the experiment (between 1–4 pm) and before sacrifice and tumor resection. All mice were fasted for 2 hours before blood collection to stabilize blood glucose levels. The mice were anesthetized with isoflurane (Halocarbon, NJ), and the blood was collected from the heart into heparinized tubes. The blood was centrifuged at 1,500 × g for 10 min, and the plasma was collected and stored at -80°C until assayed. Plasma glucose and β-OHB concentrations were measured spectrophotometrically using the StanBio^® ^Enzymatic Glucose Assay (1075-102) (StanBio Laboratory, Boerne, TX, USA) and a modification of the Williamson *et al*., enzymatic procedure [47], respectively. For blood ketone body analysis, we measured only β-OHB levels because this is the major blood ketone body in plasma [48,49].

### Histology

Tumor samples were fixed in 10% neutral buffered formalin (Sigma, St. Louis, MO) and embedded in paraffin. Tumors were sectioned and examined by light microscopy. For the histological studies, treatment was initiated as described above and was continued for 7 days to prevent tumor-associated tissue distortion.

### Factor VIII Staining and Microvessel Analysis

Tumor sections were incubated with trypsin at 37°C for 30 min after deparaffinization, rehydration, and washing as we described recently [10,11]. Briefly, the sections were quenched with 0.3% H_2_O_2_-methanol for 30 min and then blocked with 10% normal goat serum in phosphate buffer plus albumin (PBA) that contained 100 ml of 0.01 M phosphate and 0.9% sodium chloride (pH 7.4) with 1.0 g of bovine serum albumin and 0.1 ml of Tween 20. The sections were treated with rabbit polyclonal antibody directed against human factor VIII-related antigen (Dako Corp., Carpinteria, CA; 1:100 dilution with PBA) followed by a biotinylated anti-rabbit IgG at 1:100 dilution (Vector Laboratories, Inc., Burlingame, CA). The sections were then treated with avidin-biotin complex followed by 3,3'-diaminobenzidine as substrate for staining according to the manufacturers directions (Vectastain Elite ABC kit; Vector Laboratories, Inc.). The sections were then rinsed three times with 0.01 M phosphate buffer with 0.9% NaCl. Sections were counterstained with methyl green and mounted. Corresponding tissue sections without primary antibody served as negative controls. Microvessel density was quantified by examining areas of vascular hot spots in high power fields (hpf, 200 ×) as previously described [50] with some modifications [51]. Briefly, sections were scanned at 40 × and at 100 × for the localization of vascular hot spots. Blood vessels were counted at 200 × in the three most non-necrotic vascular areas of the tumor. The values of the three sections were averaged for each CT-2A tumor. Branching structures were counted as a single vessel as described previously [50]. Blood vessels were not counted in the U87 tumor due to extensive hot spot areas. In this case, only the tumor sections were shown for qualitative analysis.

### Semi-quantitative RT-PCR

Total RNA was isolated from homogenized tissue using TRIzol Reagent (Invitrogen, La Jolla, CA), following the manufacturers protocol. Spectrophotometric measurements at 260 and 280 nm determined RNA concentration and purity. Single-strand cDNA was synthesized from total RNA (3 μg) by using oligo (dT) primers (Promega, Madison, WI) in a 20-μl reaction with Moloney murine leukemia virus reverse transcriptase (M-MLV RT; Promega) according to the manufacturers protocol. cDNA (3 μl) was used for PCR amplification of specific regions of the β-OHBDH, SCOT, and β-actin genes. Primer sequences and amplicon information for the mouse and the human β-actin, β-OHBDH, and SCOT genes can be viewed at NCBI (National Center for Biotechnology Information, PubMed) using the accession numbers EF095208-EF095213. Gradient PCR was performed to obtain optimal primer annealing temperatures as previously described [52]. In order to determine the optimal linear range for semi-quantitative RT-PCR, PCR was performed at increasing cycle numbers. PCR amplification was performed with Taq DNA polymerase (Promega) using similar protocols for each gene. PCR products (10 μl) were separated on 1.0 % agarose gels containing ethidium bromide, visualized with UV light, and analyzed using 1D Kodak Software (Eastman Kodak Co., Rochester, NY).

### Statistical analysis

The one-way analysis of variance (ANOVA) was used to analyze body weight, tumor growth, plasma glucose, and β-OHB levels [53]. The Fishers PLSD test was used to calculate two-sided pair-wise comparison among different test groups by use of Statview 5.0. In each figure, n designates the number of individual mice analyzed. Error bars in the figures are expressed as mean ± SEM. Survival was computed and plotted according to the nonparametric Kaplan-Meier analysis, and comparison of control and treated groups was made using the log-rank test [53].

## Results

The objective of this study was to determine if the KetoCal^® ^ketogenic diet could be therapeutic against experimental mouse and human brain tumors when administered in restricted amounts according to recommendations for treatment of children with refractory epilepsy. In contrast to other ketogenic diet formulations (lard-based or medium chain triglyceride diets), which are not standardized or commercially available, KetoCal^® ^is a nutritionally complete medical food for children that would be widely available for alternative indications such as malignant brain cancer. Because we previously showed that CR of the high carbohydrate standard diet has anti-tumor and anti-angiogenic effects against the CT-2A and U87 experimental brain tumors [10,11], we did not include a hypocaloric group for the high carbohydrate diet in this study. Consequently, the restricted KetoCal^® ^mouse groups were compared to mouse groups receiving either the standard chow diet or the KetoCal^® ^diet in unrestricted amounts.

### Body weight and diet tolerance

Body weights remained similar in the unrestricted standard diet fed group (SD-UR) and in the unrestricted KetoCal^® ^diet fed group (KC-UR) throughout the study despite major differences in the caloric content and composition of the diets (Table 1 and Figure 1A and 1B). The restriction of KetoCal^® ^in the KC-R group was according to the manufacturers recommendation for the management of childhood epilepsy (Nutricia North America). This involved administration of 65–70% recommended daily allowance of calories or an approximate 30–35% calorie restriction. This degree of dietary restriction gradually produced an approximate 20–23% body weight reduction by 11 days post i.c. tumor implantation in the B6 and SCID KC-R groups. No signs of vitamin or mineral deficiency were observed in the KC-R mice based on standard criteria in mice [54]. Indeed, physical activity and grooming behavior was noticeably greater in the KC-R groups than in the SD-UR and KC-UR groups. These findings indicate that KetoCal^® ^was well tolerated in tumor-bearing B6 and SCID mice and, when given in restricted amounts, produced noticeable improvement in health and vitality.

### Influence of diet on tumor growth and mouse survival

**Figure 2:**
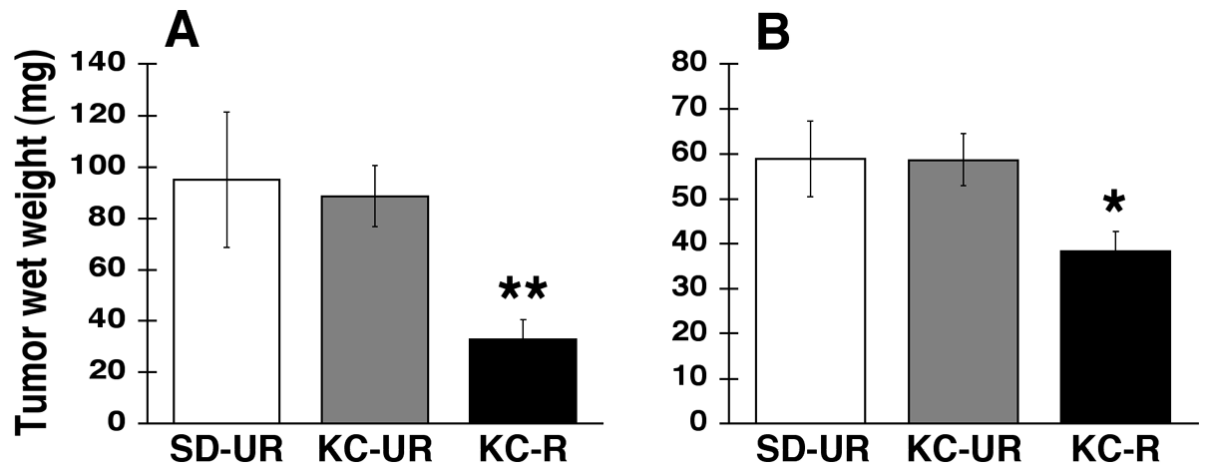
Influence of diet on the intracerebral growth of the CT-2A (A) and the U87 (B) brain tumors. The asterisks indicate that the tumor weights of the KC-R groups differed from those of the SD-UR groups at the P < 0.03 *, and the P < 0.01 ** level. Other conditions are the same as shown in Figure 1.

**Figure 3:**
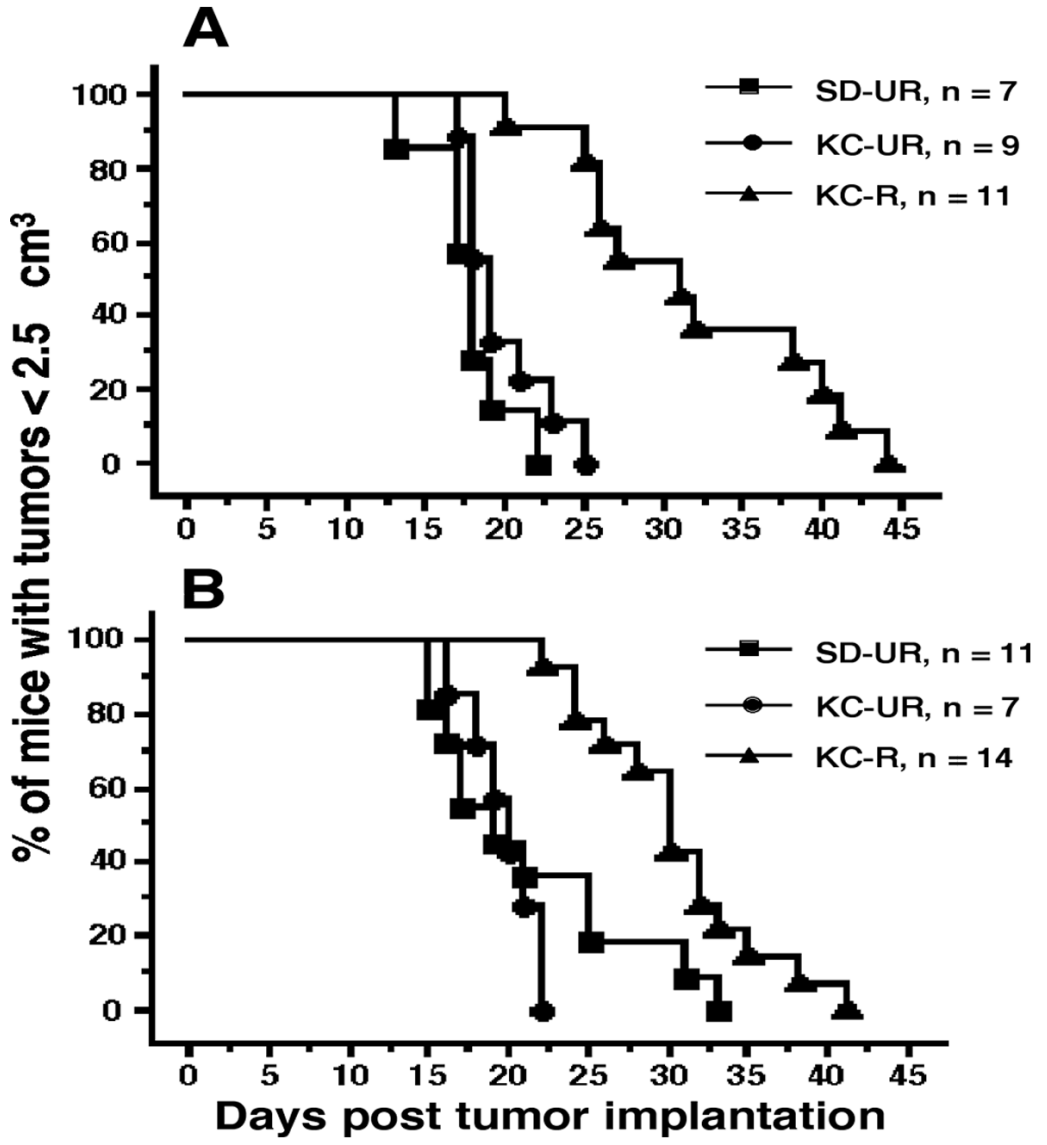
Influence of diet on the percentage of mice with the CT-2A (A) or the U87 (B) brain tumors < 2.5 cm^3 ^in size. Data are expressed as Kaplan-Meier survival curves. Survival was significantly longer in the mice on KC-R diet (▴) than on either the SD-UR or KC-UR diets P < 0.01.

Intracerebral growth of the CT-2A and U87 tumors was rapid in both the SD-UR and KC-UR groups, but growth was reduced by approximately 65% and 35% in the KC-R groups, respectively (Figure 2A and 2B). These reductions in tumor weight exceeded the reductions in body weight. It is important to mention that all implanted CT-2A and U87 tumors grew in the KC-R groups, indicating that restricted feeding did not prevent tumor "take" but significantly reduced the intracerebral growth rate. Survival, assessed as the time required for subcutaneous tumor nodules to reach 2.5 cm^3^, was significantly longer in the KC-R groups than in the SD-UR or KC-UR groups (Figure 3A and 3B). No significant differences in survival were found among tumor-bearing mice in the SD-UR or the KC-UR groups. These findings indicate that KetoCal^®^, administered in recommended restricted amounts, significantly reduced tumor growth and extended survival in mice bearing either the mouse CT-2A or human U87 brain tumors.

### Influence of diet on plasma glucose and β-OHB levels

**Figure 4:**
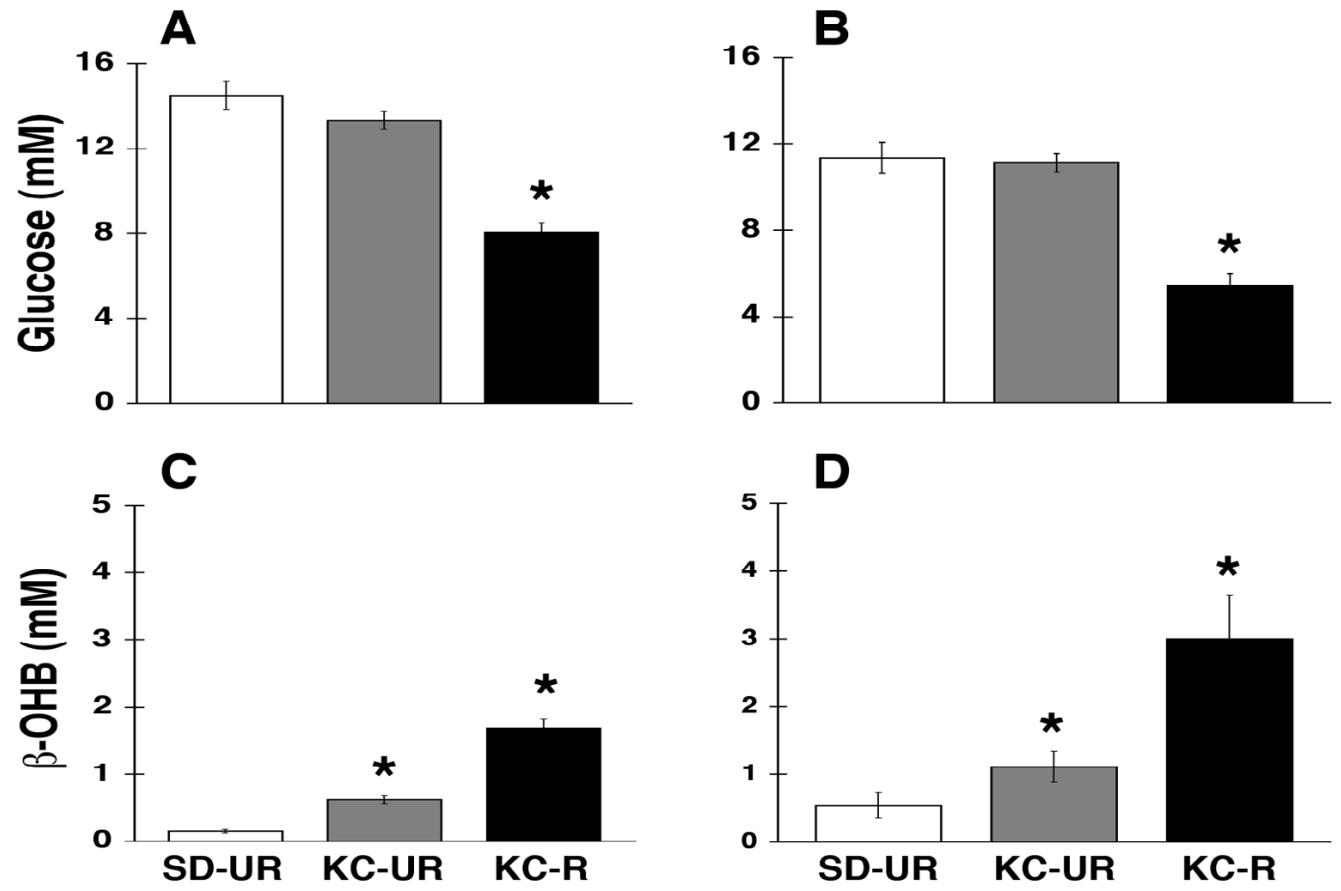
Influence of diet on plasma glucose and β-OHB levels in B6 mice bearing the CT-2A astrocytoma (A and C), or in SCID mice bearing the human U87 glioma (B and D). Values are expressed as mean ± SEM (n = 12–14 mice per group) and the asterisks indicate that the values differ from the control SD-UR group at the at the P < 0.01 level.

A transition from glucose to ketone bodies for energy under caloricaly restricted conditions is known to inhibit brain tumor growth through multiple integrated systems [5,9-11]. Plasma glucose levels were significantly lower in the KC-R mouse groups than in the SD-UR or the KC-UR groups (Figure 4A and 4B). Plasma glucose levels, however, were similarly high in both UR-fed mouse groups. These findings are consistent with our previous studies in mice showing that the KD does not lower plasma glucose levels when administered in unrestricted amounts [8,18]. In contrast to glucose levels, circulating β-OHB levels were 2 to 3-fold greater in the KC-UR groups than in the SD-UR groups (Figure 4C and 4D). Interestingly, β-OHB levels were 5 to 9-fold greater in the KC-R groups than in the SD-UR groups. These findings are also consistent with our previous studies in mice showing that circulating β-OHB levels are greater under restricted than unrestricted dietary conditions [8,18,55,56].

### Influence of diet on brain tumor vascularity

**Figure 5:**
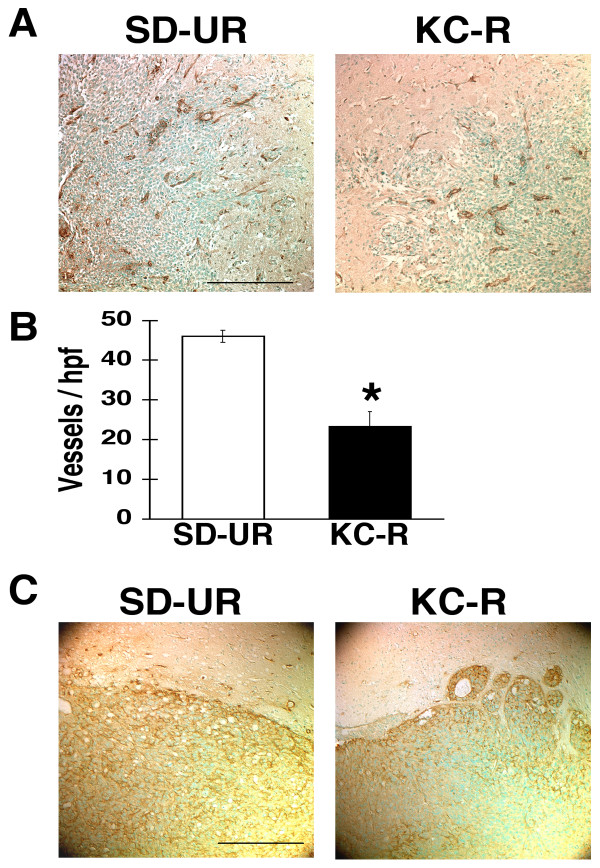
Influence of diet on vascularity in the CT-2A (A and B) and the U87 (C) brain tumors grown orthotopically in B6 or SCID mice, respectively. Vessels were stained with the Factor VIII antibody and each stained section was representative of the entire tumor. For quantitative analysis of vessels in the CT-2A tumor, microvessel density was expressed as the number of vessels/200 × high power field (hpf). The values are expressed as the mean of three independent samples with error bars representing SEM. The asterisks indicate that the number of vessels/hpf was significantly less in the KC-R group than in the SD-UR group at P < 0.05. The scale bar represents 250 μm.

To determine if KetoCal^® ^influenced angiogenesis, we used Factor VIII immunostaining to examine blood vessel densities in the CT-2A and U87 brain tumors. Three independent CT-2A and U87 tumors were chosen at random from each dietary group. The number of blood vessels in the CT-2A tumor was noticeably less in the KC-R group than in the SD-UR group (Figure 5A). Also, CT-2A microvessel density/high power field was significantly less in the KC-R group than in the SD-UR group (Figure 5B). As found in the CT-2A tumor, the number of blood vessels in the U87 tumor was noticeably less in the KC-R group than in the SD-UR group (Figure 5C). Due to the high density of blood vessels in the U87 tumor compared to the CT-2A tumor, it was not possible to accurately measure microvessel density/hpf in the U87 tumor. No differences in the number or density of blood vessels were observed between the SD-UR and KC-UR groups for either tumor (data not shown). These findings indicate that KetoCal^®^, administered in restricted amounts, was anti-angiogenic in the CT-2A and U87 brain tumors.

### Differential mRNA expression for β-hydroxybutyrate dehydrogenase (β-OHBDH) and succinyl-CoA: 3-ketoacid CoA transferase (SCOT) in normal brain and in brain tumor tissue

**Figure 6:**
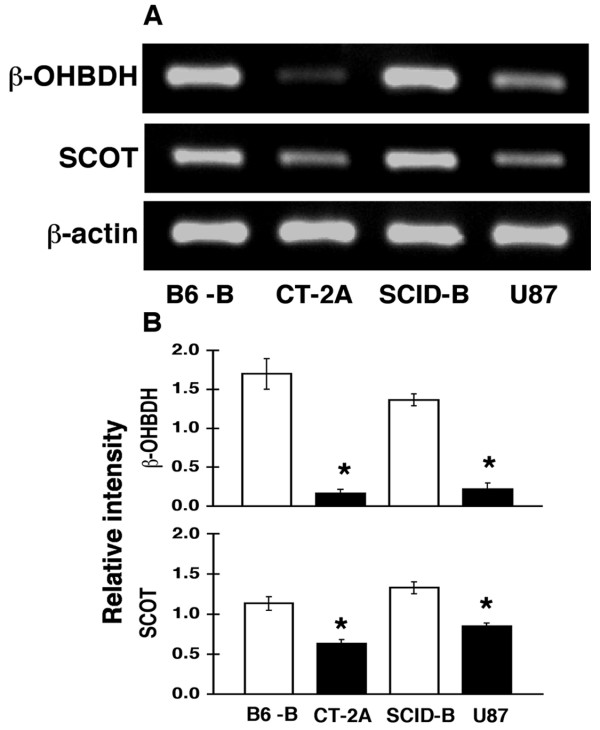
Expression of β-OHBDH and SCOT in B6 and SCID mouse brains and in CT-2A and U87 brain tumors. (A) RT-PCR was used to detect mRNA levels of β-OHBDH, SCOT, and β-actin as described in methods. Expression was evaluated in normal appearing B6 brain tissue (B6-B) contralateral to the CT-2A tumor, and in normal appearing SCID brain tissue (SCID-B) contralateral to the U87 tumor. (B) The ratio (relative intensity) of β-OHBDH and SCOT expression to β-actin expression. The values are expressed as the mean of three-four independent samples with error bars representing SEM. The asterisks indicate that the ratio was significantly less in the tumor tissue (solid bars) than in the normal brain tissue (open bars) at P < 0.01.

β-OHBDH and SCOT are required for the metabolism of ketone bodies for energy in brain mitochondria [13,20,21]. We used semi-quantitative RT-PCR to compare β-OHBDH and SCOT mRNA levels in the CT-2A and U87 tumor tissue grown in the right cerebral hemisphere with those levels in the normal appearing brain tissue of the contralateral left hemisphere (Figure 6A and 6B). β-OHBDH and SCOT mRNA levels were significantly lower in the CT-2A and the U87 tumor tissue than in the normal appearing contralateral brain tissue. No differences in β-OHBDH and SCOT mRNA levels were found between normal appearing brain tissue from tumor-bearing mice and brain tissue from non-tumor-bearing mice (data not shown). These findings suggest that the CT-2A and the U87 tumor cells are less capable than the normal mouse brain cells in using ketone bodies for energy.

## Discussion

We found that KetoCal^®^, a nutritionally balanced and commercially available ketogenic diet for children with epilepsy, significantly reduced the orthotopic growth and the vascularity of the mouse astrocytoma (CT-2A) and the human glioma (U87). Moreover, KetoCal^® ^significantly prolonged survival in the tumor-bearing mice. It is important to mention that the anti-angiogenic and growth inhibitory effects of KetoCal^® ^were observed only when the diet was administered in restricted amounts but were not seen when the diet was administered *ad libitum*, or in unrestricted amounts. These findings support previous observations that restriction of dietary calories has powerful anti-angiogenic and anti-inflammatory effects against cancer, including brain cancer [9-11,51,57,58]. Reduced caloric content lowers circulating glucose levels as we found in this study and in our previous studies [10,11]. Indeed, tumor growth is more strongly correlated with circulating glucose levels than with circulating ketone body levels [8]. The reduction in glucose levels following restriction of dietary calories largely accounts for why tumors grow minimally on either restricted ketogenic diets or on restricted high carbohydrate standard diets. Restriction of calories in humans may be difficult to achieve, however, due to issues of compliance. Compliance may be better with KetoCal^® ^as this diet was designed for managing refractory human epilepsy under calorically restricted conditions. CR, however, is not directly comparable in mice and humans. For example, a 40% CR diet in mice is comparable to therapeutic fasting in humans, which can be difficult for many people [30]. In addition to reducing circulating glucose levels, a restriction of total calories also reduces potential adverse effects of the high fat content of the diet since energy homeostasis is maximized under CR regardless of caloric origin [8,30]. The restricted KetoCal^® ^diet should therefore be easier to implement than therapeutic fasting for brain cancer patients.

Although we previously showed that CR of a high carbohydrate standard diet or of a rodent ketogenic diet similarly reduce blood glucose levels, which tumor cells depend upon for survival [10,11], our findings in this study showed that administration of KetoCal^® ^under restricted conditions was more effective in elevating circulating ketone bodies than was administration under unrestricted conditions. This is important since mild ketosis, under conditions of reduced glucose availability, is essential for enhancing the bioenergetic potential of normal brain cells [5,17,18]. Additionally, ketone bodies may directly protect normal neurons and glia from damage associated with aggressive tumor growth through a variety of neuroprotective mechanisms [59-64]. In contrast to most conventional brain tumor therapies, which indiscriminately target both normal cells and tumor cells, CR and particularly restricted ketogenic diets such as KetoCal^® ^are the only known therapies that can target brain tumor cells while enhancing the health and vitality of normal brain cells [5,29]. In this regard, the calorically restricted ketogenic diet for brain cancer management stands apart from all conventional therapeutic approaches.

Previous studies indicate that many tumors including brain tumors are largely unable to metabolize ketone bodies for energy due to various deficiencies in one or both of the key mitochondrial enzymes, β-OHBDH and SCOT [19,21,65-68]. Our gene expression results in the mouse CT-2A and the human U87 brain tumors are consistent with these previous findings in other tumors and also support the mitochondrial defect theory of cancer [5,23,24,69]. The deficiencies in these enzymes, however, are important for tumor management only under calorically restricted conditions when glucose levels are reduced and when cells would require ketone bodies for energy. This is most evident from the analysis of tumor growth in the unrestricted KetoCal^® ^groups where growth was rapid despite mild ketosis. This is attributed to the maintenance of high glucose levels, which the tumor cells will use for energy in preference to ketone bodies due to their dependency on glycolysis. However, when glucose becomes limited, as occurs under CR, the SCOT and β-OHBDH deficiencies would prevent tumor cells from using ketones as an alternative fuel thus metabolically isolating the tumor cells from the normal cells. We suggest that the genes for these enzymes could be useful markers for screening brain tumors and other tumor types that may be responsive to therapy using restricted KetoCal^® ^or other restricted ketogenic diets. Further studies will be necessary to test this interesting possibility.

Long-term management of malignant brain cancer has been difficult in both children and adults. This has been due in large part to the unique anatomical and metabolic environment of the brain that prevents the large-scale resection of tumor tissue and impedes the delivery of chemotherapeutic drugs. Further, significant neurological abnormalities are often observed in long-term brain tumor survivors [70-72]. In light of the differences in energy metabolism between normal brain cells and brain tumor cells, we recently proposed an alternative approach to brain cancer management based on principles of evolutionary biology and metabolic control theory [5]. Specifically, the genomic and metabolic flexibility of normal cells, which evolved to survive under physiological extremes, can be used to target indirectly the genetically defective and less fit tumor cells. The results of this study using restricted KetoCal^® ^as a therapy for experimental brain cancer provide direct support for this alternative approach to brain cancer management. It should be recognized that this alternative therapeutic approach may not be restricted only to brain cancer, but could also be effective for any cancer types containing genetically compromised and metabolically challenged tumor cells. Moreover, KetoCal^® ^will be more effective in managing brain tumors in humans than in managing brain tumors in mice since prolonged caloric restriction can be tolerated better in humans than in mice due to intrinsic differences in basal metabolic rate [30].

Although Nebeling and co-workers were successful in managing childhood astrocytoma with a medium-chain triglyceride ketogenic diet [29,73], this KD formulation can be difficult to implement, is not standardized, and can produce some adverse effects as previously observed in children taking the diet for epilepsy management [32-34]. It is noteworthy that the children treated in the Nebeling study also expressed reduced blood glucose levels [29]. When administered in restricted amounts, KetoCal^® ^may have greater therapeutic efficacy with fewer side effects than medium-chain triglyceride or lard-based KDs. Additionally, KetoCal^® ^would eliminate or reduce the need for antiepileptic drugs for brain tumor patients since KetoCal^® ^was designed originally to manage refractory epileptic seizures. Adjuvant steroidal medications, which are often prescribed to brain tumor patients and which can produce severe adverse effects, might also be reduced under the restricted KetoCal^® ^diet since glucocorticoid levels increase naturally under calorically restricted conditions [74-76]. Ketogenic diets and calorically restricted diets can also antagonize cancer cachexia [9,11,77]. These observations, considered together with the anti-angiogenic effects of the diet, suggest that the restricted KetoCal^® ^diet can manage brain tumors through multiple integrated systems.

Guidelines for the implementation of KetoCal^® ^and other calorie restricted KDs in younger and older patients have been described previously [5,29,34,73]. KetoCal^® ^could be administered to patients with brain tumors at medical centers or clinics currently using the ketogenic diet for managing epilepsy. Based on our findings in mice and those of Nebeling and co-workers in humans, initiation of randomized clinical trials are warranted to determine whether KetoCal^® ^is effective for the long-term management of malignant brain cancer and possibly other glycolysis dependent cancers [78]. This is especially pertinent to patients with glioblastoma multiforme, an aggressive brain tumor type for which few effective therapeutic options are available. While KetoCal^® ^was formulated for managing childhood seizures, it is likely that new KD formulations can be designed with nutritional and caloric compositions more appropriate for managing brain tumors [5,78]. This could also involve the use of low glycemic diets, which are effective in maintaining low circulating glucose levels and are easier to implement than some ketogenic diets [79,80]. Additionally, the diets could be combined with specific drugs to further enhance therapeutic efficacy. As a cautionary note, the calorically restricted KD would not be recommended for those few individuals with fasting intolerance due to defects, either inherited or drug-induced, in carnitine or fatty acid metabolism [49]. Our results in mouse and human brain tumor models suggest that the restricted KetoCal^® ^diet will be an effective alternative therapy for managing malignant brain cancer in humans and should be considered as either a first line or adjuvant therapeutic option.

## Conclusion

The results indicate that KetoCal^® ^administered in restricted amounts has anti-tumor and anti-angiogenic effects in experimental mouse and human brain tumors. Furthermore, genes for ketone body metabolism will be useful for screening brain tumors that could be targeted with KetoCal^® ^or other calorically restricted high fat low carbohydrate diets. This preclinical study in mice indicates that the restricted KetoCal^® ^diet should be an effective alternative therapeutic option for managing malignant brain cancer in humans.

## List of Abbreviations

The abbreviations used are: CT-2A, mouse astrocytoma; U87, human malignant glioma (U87-MG); CR, Caloric restriction; β-OHB, β-hydroxybutyrate; β-OHBDH, β-hydroxybutyrate dehydrogenase; SCOT, succinyl-CoA: 3-ketoacid CoA transferase; KD, Ketogenic Diet; B6, C57BL/6J; SCID, BALBc/J-severe combined immunodeficiency; intracerebral, i.c.; SD, standard high carbohydrate mouse chow diet; SD-UR, standard diet fed *ad libitum*, or unrestricted; KC-UR, KetoCal^® ^diet fed *ad libitum*, or unrestricted; KC-R, KetoCal^® ^diet restricted.

## Competing interests

The author(s) declare that they have no competing interests.

## Authors' contributions

WZ carried out all described methods and drafted the manuscript. PM helped with the design of the study and data analysis. MAK participated in the design of the primers, sequence and data analysis. WTM participated in the feeding of the mice, tumor implantation, and data analysis. JGM carried out the ketone assays, and participated in data and statistical analysis. TNS conceived the study, participated in its design and coordination and helped prepare the manuscript. All authors read and approved the final manuscript.

## Note

Data deposition footnote: Primer sequences and amplicon information for the mouse and the human β-actin, β-OHBDH, and SCOT genes can be viewed at NCBI (National Center for Biotechnology Information, PubMed) using the accession numbers EF095208-EF095213.
